# Effective usage of a clearance check to avoid a collision in Gamma Knife Perfexion radiosurgery with the Leksell skull frame

**DOI:** 10.1093/jrr/rru057

**Published:** 2014-06-24

**Authors:** Hisato Nakazawa, Takahiko Tsugawa, Yoshimasa Mori, Masahiro Hagiwara, Masataka Komori, Chisa Hashizume, Yuta Shibamoto, Tatsuya Kobayashi

**Affiliations:** 1 Department of Radiological Sciences, Nagoya University Graduate School of Medicine, 1–1–20 Daikominami, Higashi, Nagoya, Aichi 461–8673, Japan; 2 Nagoya Radiosurgery Center, Nagoya Kyoritsu Hospital, Nagoya, Japan; 3 Department of Radiology and Radiation Oncology, Aichi Medical University, Nagakute, Japan; 4 Department of Radiology, Nagoya City University Graduate School of Medical Sciences, Nagoya, Japan

**Keywords:** collision warning, simulation, skull frame placement, stereotactic localization, stereotactic radiosurgery, Gamma Knife

## Abstract

Skull frame attachment is one of the most significant issues with Gamma Knife radiosurgery. Because of the potential for suffering by patients, careful control of the frame position is required to avoid circumstances such as collision between the frame or the patient's head and the collimator helmet, and inaccessible target coordinates. This study sought to develop a simulation method to find the appropriate frame location on the patient's head by retrospective analysis of treatment plans for brain metastasis cases. To validate the accuracy of the collision warning, we compared the collision distance calculated using Leksell GammaPlan (LGP) with actual measured distances. We then investigated isocenter coordinates in near-collision cases using data from 844 previously treated patients and created a clearance map by superimposing them on CT images for just the frame, post and stereotactic fiducial box. The differences in distance between the simulation in LGP and the measured values were <1.0 mm. In 177 patients, 213 lesions and 461 isocenters, there was a warning of one possible collision. The clearance map was helpful for simulating appropriate skull frame placement. The clearance simulation eliminates the psychological stress associated with potential collisions, and enables more comfortable treatment for the patient.

## INTRODUCTION

**Fig. 1.**
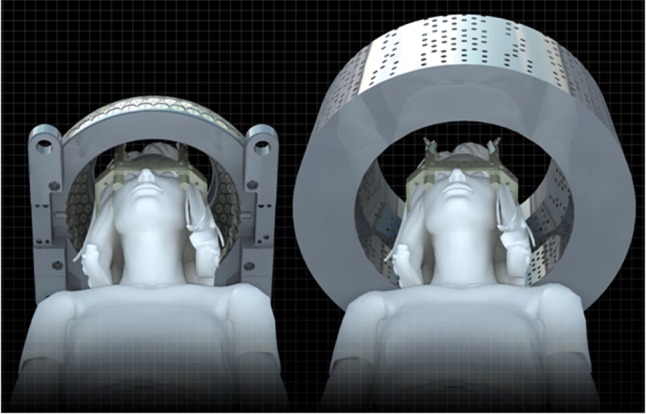
The collimator helmet shape of both Model C (left) and Perfexion (PFX) (right). The accessible space in PFX is much wider than that in Model C. Note that the treatable range in Model C is as follows: *x* = 37.5 to 162.5 mm, *y* = 30 to 170 mm, and *z* = −11 to 142 mm, while that in PFX is *x* = 20 to 180 mm, *y* = 10 to 190 mm, and *z* = −820 to 167 mm. This picture was provided by Elekta.

Gamma Knife (GK) (Elekta, Tokyo) stereotactic radiosurgery (SRS) is used for intracranial lesions. The GK unit employs a rigid Leksell skull frame (Elekta, Tokyo) to ensure high positional accuracy of 3D converging beams from multiple ^60^Co sources focused on the target. The latest version of GK, Perfexion (PFX), differs from the previous models, B and C, in many ways [1–3]. The most important refinement for clinical treatment is expansion of the treatment range. Compared with the hemispherical-shaped collimator helmet in the Model C system, the cone-shaped helmet in the PFX system has increased the available access in stereotactic space (Fig. 1). In clinical treatments with Model C, we often experienced cases in which a planned beam could not be delivered because of a collision between the collimator helmet and the patient's skull or the frame, including the post and screw. In the procedure with PFX, it is still important to pay attention to frame placement, though almost all targets in the brain may be reached when the frame is placed on the center of the patient's head. This study aimed to develop a method to simulate appropriate skull frame placement to allow access to the various intracranial targets before actual treatment by retrospective analysis of 1096 cases. This method is thought to be particularly useful for patients with multiple brain metastases.

## MATERIALS AND METHODS

The Research Ethics Board of Nagoya University Graduate School of Medicine approved this clinical study (Approval No. 12–310).

### Skull frame placement on the head and radio-surgical technique

The skull frame is attached by a neurosurgeon using information from skull radiographs after confirming the location of the target detected using diagnostic contrast-enhanced computed tomography (CT) and magnetic resonance imaging (MRI) taken prior to GK treatment. The default settings of a long post and screw, which are parts of the frame, are as indicated below: anterior post height: 123 mm, posterior post height: 85 mm, length of the screw head protruding outward from the post: <10 mm. Except for the case when scheduled targets are located in the extreme periphery of the brain, the skull frame is attached to approximately the center of the patient's head. After the skull frame attachment, contrast-enhanced MRI and CT are acquired and exported to the Leksell GammaPlan (LGP) treatment-planning device. The Leksell stereotactic coordinates system is defined using visible marker positions scanned using a dedicated fiducial box attached to the frame and determines positional relationships based on the frame. Radio-surgical planning is performed to achieve an appropriate dose and dose distribution for the target in LGP. When the completed treatment plan from the LGP cannot actually be performed on the GK unit because of collisions, an additional skull frame placement is required. If this happens, the patient must endure more pain and suffering associated with the frame attachment, and additional stereotactic imaging and planning are required, subjecting the patient to a tremendous burden. After verifying that the treatment plan is free from the possibility of collision, actual irradiation is carried out.

The LGP system informs the surgeon of the possibility of a collision by a collision warning, which occurs when the distance between the frame, post and screw and collimator helmet is less than a preset level. The preset levels are 12.0 mm for the frame, post and screw, and 19.0 mm for the patient's skull when a dedicated skull helmet for creating skull contours is used, and the collision-warning coordinates are confirmed with a collision check tool before GK treatment delivery. The collision check tool simulates collision with the collimator helmet inside the PFX machine. The positional relationship between the collision check tool and the object of collision corresponds to that between the collimator helmet and the object of collision. The structural coincidence of both combinations is ensured by the vendor (Elekta) (undisclosed data).

### Validation of collision warning in GK treatment plan

To evaluate the accuracy of collision warnings in LGP, we compared the collision distance calculated in LGP with actual measured distance. The collision detection was verified using a set of only the frame, posts and screws, because the object of collision was not the skull but a post or screw in most of the clinical cases. The frame–post configuration was set at the default setting. The fiducial box for the coordinate system was then attached to the frame, and a CT scan was performed and loaded into LGP. In LGP, a spherical skull-contouring model with a 100-mm radius was used as the default setting. In LGP, to cover the possible whole range of collision warnings, 40 isocenters were placed at a range of positions, such as frontal, cerebellar, occipital and parietal regions, where collision alerts occur frequently in clinical cases, so that collision warnings ranging from 1.0 to 12.0 mm appeared. The collision check for each isocenter was confirmed with a dedicated clearance check tool provided by the manufacturer (Elekta) when the set of the frame and the tool were docked with the couch of PFX, and the couch was moved to each isocenter position. The distance between the tool and the object of collision was measured in 0.5-mm increments using a ruler. The calculated distances were compared with the actual measured values. The ruler measurement was carefully double-checked by two technicians experienced with PFX. This procedure was conducted only for gamma angles of 90°, which are available for almost all cases of PFX. In LGP, the orientations of the axes in the stereotactic coordinates system were *x* as right–left direction, *y* as posterior–anterior direction, and *z* as superior–inferior direction.

### Retrospective evaluation of clearance warning coordinates on LGP

To simulate appropriate skull frame placement enabling access to various intracranial targets, we investigated collision-warning coordinates using treatment-planning information from previously treated patients. The 1096 cases treated by PFX between October 2010 and July 2012 were enrolled in this study. Metastatic brain tumors from other organs totalled 844 cases and represented more than 77% of the entire cases. The 3D coordinates of collision warnings were collected and then superimposed on CT images consisting of only the frame, posts and stereotactic indicator box. All of the collision warning values were in the plus range. The distribution profile of collected data was approximately elliptical in shape, because the collimator shape of PFX is a circular cylinder (Fig. 1). To approximate this elliptic shape, we used the least-squares method. The formula for an ellipse is mathematically given by the expression:

$$\begin{array}{c} {\left\{\frac{\left(X-X_{0}\right)\cos\theta +\left(Y-Y_{0}\right)\sin\theta }{a}\right\}}^{2}\\ \;+{\left\{\frac{-\left(X-X_{0}\right)\sin\theta +\left(Y-Y_{0}\right)\cos\theta }{b}\right\}}^{2}=1. \end{array}$$

*X_0_* and *Y_0_* are the center coordinates of the *x* and *y* axes of the ellipsoid, respectively, *θ* is the slope of the ellipsoid, and *a* and *b* are half the length of the major (*x*) and minor (*y*) axes. Because there is no way to directly display this approximated curve in LGP, we placed isocenters using coordinates obtained by this formula (*x* range: 40–160 mm, incremented by Δx = 1 mm) and described the area of clearance warning on the monitor using the isodose line function in the LGP software. The size of the area was changed by selecting the collimator size and by adjusting the percentage of isodose. A clearance map was created from plotted data of the collision warnings to make the collision range clear. When all targets are inside the clearance range, theoretically they can be accessed in GK surgery. The cranial–caudal direction was not considered in this study (details described below).

## RESULTS

### Validation of collision warning in GK treatment plan

Table 1 shows the results of collision-warning evaluation for the 40 isocenters using a set of the frame, post and screw. The difference between the clearance values calculated in LGP and those measured using the ruler was <1.0 mm.

**Table 1.** Validation of collision-warning for the 40 isocenters using a set of frame, post and screw

Isocenter number	*x* (mm)	*y* (mm)	*z* (mm)	Collision position	Calculation in LGP (mm)	Measurement (mm)	Deviation (mm)
1	49.1	37.8	138.4	AL	1.0	1.0	0.0
2	76.9	33.1	139.1	AL	1.0	1.5	0.5
3	89.7	32.1	139.3	AL	1.5	2.0	0.5
4	100.0	31.9	139.5	AR	2.0	2.5	0.5
5	57.2	38.8	148.0	AL	1.0	1.5	0.5
6	54.3	39.8	146.4	AL	1.0	1.5	0.5
7	54.3	38.8	139.6	AL	1.0	1.5	0.5
8	163.0	50.8	138.0	AR	2.5	3.0	0.5
9	145.9	40.1	137.7	AR	3.0	4.0	1.0
10	137.3	38.2	137.7	AR	3.5	3.5	0.0
11	121.1	35.6	137.8	AR	4.0	4.5	0.5
12	112.3	34.9	137.7	AR	4.5	5.0	0.5
13	104.3	34.6	137.5	AR	5.0	5.0	0.0
14	46.3	36.3	62.6	AL	1.0	1.5	0.5
15	44.7	37.4	62.6	AL	1.0	1.5	0.5
16	44.6	36.4	53.7	AL	1.0	2.0	1.0
17	123.6	20.7	63.2	AR	2.0	3.0	1.0
18	59.2	165.8	99.3	PL	1.0	1.5	0.5
19	61.0	166.9	99.3	PL	1.0	1.5	0.5
20	59.2	166.9	86.9	PL	1.0	1.5	0.5
21	143.3	164.3	99.2	PL	1.0	1.5	0.5
22	20.1	68.5	107.6	PR	3.0	4.0	1.0
23	179.8	69.9	101.6	AL	4.0	5.0	1.0
24	171.0	57.6	101.5	AR	2.0	2.5	0.5
25	144.7	35.3	63.6	AR	5.0	5.5	0.5
26	58.8	42.0	109.8	AL	9.0	9.0	0.0
27	155.3	50.0	137.6	AR	7.0	7.5	0.5
28	65.7	158.0	99.6	PL	11.0	12.0	1.0
29	58.3	152.2	99.5	PL	12.0	12.0	0.0
30	70.6	162.0	99.3	PL	10.0	10.0	0.0
31	141.9	159.2	99.6	PR	6.0	7.0	1.0
32	128.0	165.0	99.3	PR	8.0	9.0	1.0
33	150.0	154.2	99.4	PR	5.5	6.0	0.5
34	143.0	157.9	92.6	PR	6.5	7.0	0.5
35	142.3	157.2	90.3	PR	7.5	8.0	0.5
36	39.4	60.8	99.1	AL	11.5	12.0	0.5
37	46.4	50.6	99.4	AL	8.5	9.5	1.0
38	34.6	62.6	95.5	AL	9.5	10.0	0.5
39	176.1	75.8	100.9	AR	10.5	11.5	1.0
40	144.7	35.3	63.6	AR	5.0	5.0	0.0

The origin of the Leksell coordinate system (the point where *x*, *y* and *z* are numerically zero) is located outside the coordinate frame at a point that is superior, lateral and posterior to the coordinate frame on the patient's right side. The orientations of the axes in the stereotactic coordinates system are *x* as right–left direction, *y* as posterior–anterior direction, and *z* as superior–inferior direction. The only objects generating collision warnings are the post and screw. AR = anterior right, AL = anterior left, PR = posterior right, PL = posterior left.

### Retrospective evaluation of clearance-warning coordinates on LGP

**Fig. 2.**
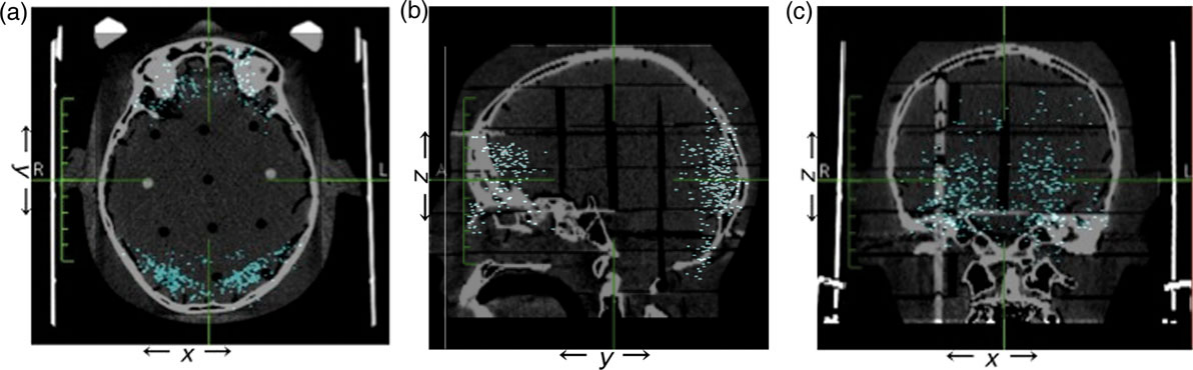
Axial (**a**), sagittal (**b**), and coronal (**c**) computed tomography images on which clearance-warning coordinates obtained from 177 patients, 213 lesions and 461 isocenters treated by PFX were superimposed.

**Fig. 3.**
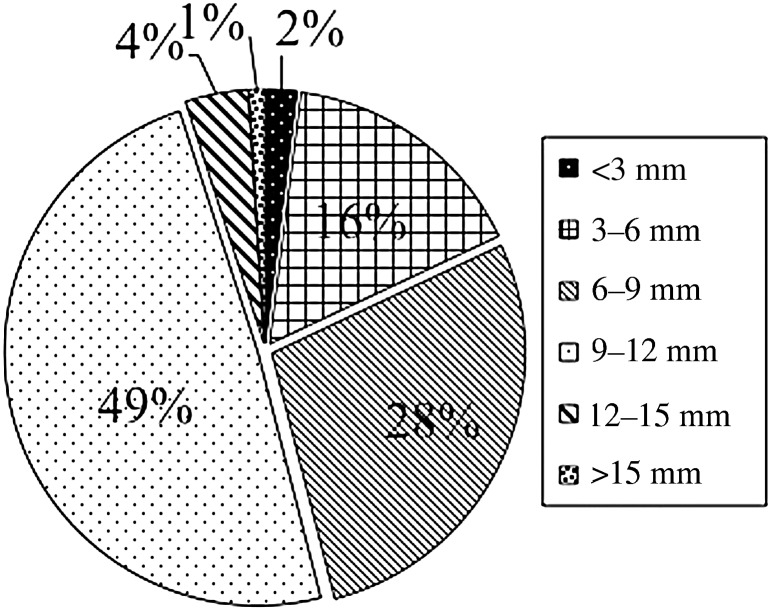
The clearance distance between the post and screw of a skull frame and the collimator helmet. The distance ranged from 9–12 mm in many cases (∼50% of all cases).

**Fig. 4.**
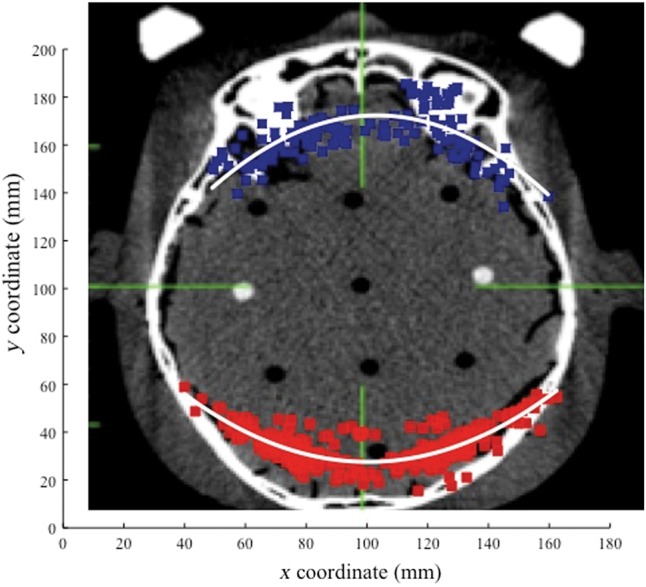
An approximated curve was calculated by the least-squares method and superimposed on an axial image.

**Fig. 5.**
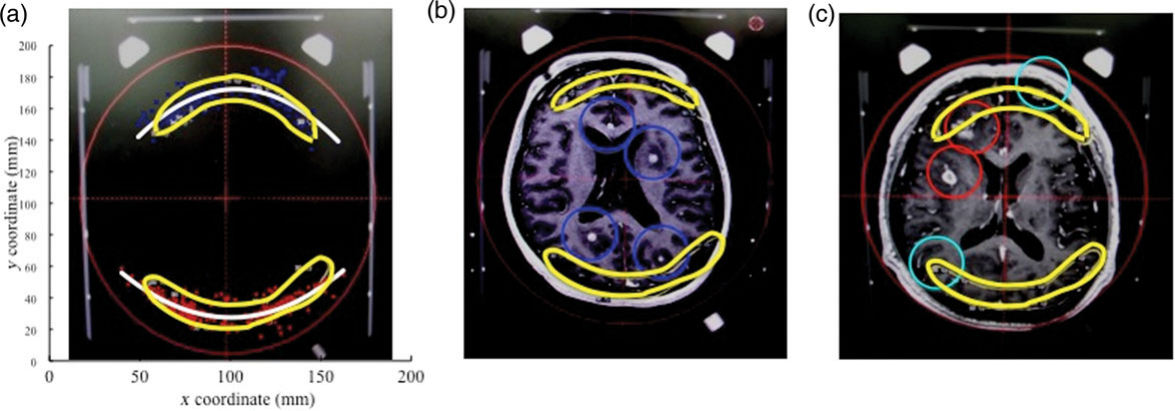
The clearance-warning area created with the approximated curve of the patient dataset. When the target is located outside this region (yellow line), it may be difficult to access the target (**a**). When the target is included in this region, all targets (blue circle) can be treated (**b**). When the target is located outside this region, it is necessary to pay attention to the possibility of collision. In these cases, the red circle could be accessible but the cyan circle may not be (**c**).

For 177 patients, 213 lesions and 461 isocenters, collision warnings were issued by LGP. Figure 2 shows the location of the 461 isocenters that caused collision warnings. The potential collision points were mainly found in the occipital lobe (261 points, 57%) and the frontal lobe (141 points, 32%). The majority of collision warnings were caused by the post and screw (443 isocenters, 96.1%). The distance between the post, screw and collimator helmet ranged from 9 to 12 mm in many cases (∼50% of all cases) (Fig. 3). In contrast, cases of <3.0 mm and >15.0 mm for collision warnings were infrequent. The center coordinates (*X_0_*, *Y_0_*) of the approximated curve obtained from these clearance plot data (Fig. 2) were 102.00 and 99.29, respectively. *θ, a* and *b* were −0.02, 52.57 and 78.63, respectively. Figure 4 is an axial image on which the approximated curve was superimposed. Figure 5a shows the clearance map (use of 80% isodose line of a 16 mm-collimator) with the approximated curve visualized in the LGP console.

## DISCUSSION

In this study, we developed a method of simulating appropriate skull frame placement and enabling access to the scheduled intracranial targets before actual treatment. The most remarkable characteristic of this method is that it can be used in the LGP treatment-planning system, which is commonly used for GK. Clinically, it performs a desirable function when treating multiple metastatic lesions located separately in the brain.

Practical software allowing simulation of frame attachment and collision checking utilizing DICOM images is already available in the Model C unit [4–6]. However, in PFX, software offering a similar function has not been reported so far. PFX does not generally need such strict control of skull frame placement because the treatment range is significantly wider. Almost all targets in the brain may be reached when the frame is placed on the center of the patient's head. However, since collision remains a possibility, especially for targets located in the periphery of the brain, it is still important to pay attention to frame placement.

Our study demonstrated a clinically useful method of ensuring appropriate skull frame placement by analyzing the distribution of collision-warning coordinates extracted from retrospective clinical cases. Because collision warnings distribute only at the anterior and posterior of the head, we ignored the cranial–caudal direction. Contrast-enhanced diagnostic CT and/or MRI without a skull frame taken prior to GK treatment are co-registered with the clearance map before actual skull frame placement, and the ideal positional relationship between the skull frame and the patient's head is determined (Fig. 5b). We determined the screw length based on the distance between post and skull calculated by simulation using a measurement tool in LGP. After that, the patient's head was placed in an appropriate position where the determined screw length in the simulation became adequate, and then the frame was attached to the patient's head. In multiple brain metastasis cases, this map can confirm that all scheduled targets are within the accessible treatment range. In particular, when two targets are located in the peripheral region of the frontal and occipital lobe, respectively, special attention has to be paid to the post height, screw length and positional relationship between the frame and the patient's head (Fig. 5c). Validation of simulations using the clearance map was performed in more than 10 cases, and all cases successfully reproduced the positional relationship between the simulated and the actual setting. The most difficult among these cases is described below.

### ‘Illustrative Case’

**Fig. 6.**
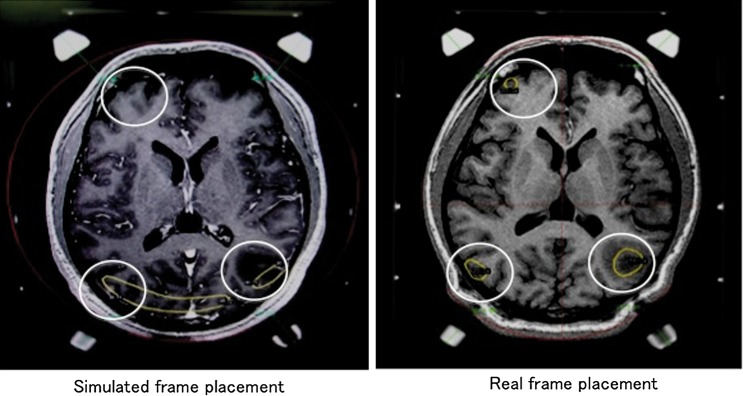
Planning of skull frame placement on an axial image for a patient with multiple brain metastases exhibited in the ‘Illustrative Case’. In order to enclose the three lesions (white circles) threatening a collision within the treatable area, the frame was adjusted to a more appropriate position using the information from the simulation.

PFX SRS was administered to a 39-year-old man with multiple brain metastases spread across a wide area of the brain (Fig. 6). Pre-treatment thin-slice (2-mm) MRIs without a skull frame (acquired at 1.5 Tesla) were loaded into the LGP workstation. Virtual simulation of the frame placement based on the clearance map was performed using the MRIs, and we checked whether the planned targets were included within the treatable field. The simulation revealed that two lesions located in the peripheral area of the right frontal lobe and right occipital lobe would be difficult to access with a single frame placement. In the simulation planning, the clearance distances were calculated by placing the isocenter for each target and were estimated to be 4.0 mm for the right frontal, 4.3 mm for the right occipital, and 11.8 mm for the left occipital targets, respectively. Therefore strict skull frame placement seemed to be required, and the frame was placed with the help of the results of this simulation and with particular attention to adjustment in the anterior–posterior direction. Stereotactic frame-fixed MRIs were scanned with a stereotactic fiducial indicator box and loaded into the same patient file in the LGP workstation. The pre-scanned frameless MRIs in association with virtual dose distributions and the isocenter position were co-registered on the stereotactic MRIs taken with the skull frame on, and then the stereotactic coordinates of the isocenter were determined. In real treatment planning, the clearance distances were 4.1 mm for the right frontal, 4.1 mm for the right occipital, and over 12.0 mm for the left occipital lesions, and the treatment was normally carried out without collisions. This simulation provided clinically useful information for actual skull frame placement.

Although the present study indicated significant benefits for a clearance map, there were some limitations to the study. First, this clearance map was created based on the default setting in our institute, and it may change for different frame–post settings. Second, a relatively small number of patients was evaluated. Further studies collecting data from more patients will be necessary to confirm our conclusions. Finally, the clearance map remains a rough indication for checking collisions in GK PFX. The clearance map was created based on collision warnings. However, the coordinates of the collision warning have some uncertainty. The coordinates of the warnings are calculated based on information such as post height. When the frame is mounted on the patient's head, the posts may bow outwards slightly as a result of the torque applied. Even though the torque is controlled by means of a torque wrench, the amplitude of the warpage may differ between cases. In our experience, an ∼3-mm margin may be required to take account of this warpage. In addition, distortion of MRI affects the coordinates of the collision warning. In our commissioning test, the distortion was <0.8 mm over the stereotactic coordinate system [7].

In conclusion, clearance simulation for frame placement eliminates operator psychological stress by determining the optimal frame–head relationship for the patient through frame positioning.
